# Photoassisted Degradation of a Herbicide Derivative, Dinoseb, in Aqueous Suspension of Titania

**DOI:** 10.1100/2012/251527

**Published:** 2012-03-12

**Authors:** Niyaz A. Mir, Malik M. Haque, Abuzar Khan, Mohd. Muneer, Colin Boxall

**Affiliations:** ^1^Department of Chemistry, Aligarh Muslim University, Aligarh 202002, India; ^2^Faculty of Science and Technology, Engineering Building, Lancaster University, Lancaster LA1 4YR, UK

## Abstract

The titanium dioxide (TiO_2_) photoassisted degradation of herbicide dinoseb has been examined in aqueous suspensions under UV light irradiation. The degradation kinetics were studied under various conditions such as substrate concentration, type of catalyst, catalyst dosage, pH, and light intensity as well as in presence of electron acceptors such as hydrogen peroxide, potassium bromate, and potassium persulphate under continuous air purging, and the degradation rates were found to be strongly influenced by these parameters. The Degussa P25 was found to be more efficient photocatalyst as compared to other photocatalysts tested. Dinoseb was found to degrade efficiently in acidic pH and all the electron acceptors studied enhanced the degradation rate. The results manifested that the photocatalysis of dinoseb followed pseudo-first-order kinetics. A qualitative study of the degradation products generated during the process was performed by GC-MS, and a degradation mechanism was proposed.

## 1. Introduction

In recent years, the release of toxic and persistent organic pollutants such as pesticides, polychlorinated biphenyls (PCBs), halogenated organic solvents, and polycyclic aromatic hydrocarbons (PAHs) into aquatic environment from industrial and wastewater treatment plants, agricultural run-off has drawn much attention and is considered one of the baffling problems facing environmental scientists today [1]. Due to their chemical stability, resistance to biodegradation, and sufficient water solubility, these organic pollutants penetrate deep into the ground water [2, 3].

Among various techniques proposed and/or being developed, heterogeneous photocatalysis has proved one of the promising techniques for complete oxidative mineralization of pollutants [4, 5]. The process is photo-induced and requires irradiation by UV-Vis light for the activation of the catalyst which is a suspension of semiconductor powder (usually metal oxide). Heterogeneous photocatalysis is a part of a family of techniques called advanced oxidation processes (AOPs) and has received much attention because of complete oxidation of pollutants, removal of inorganic compounds, heavy metals, bacteria, and viruses from water [6, 7]. Among various oxide semiconductor photocatalysts, TiO_2_ has proven to be the most suitable for widespread environmental applications due to its biological and chemical inertness, strong oxidizing power, lower cost, and long-term stability against photo corrosion and chemical corrosion [8, 9]. The photocatalyzed degradation of various organic systems employing irradiated TiO_2_ is well documented in the literature [3, 10]. The basic principles of photooxidation are well established [11, 12].

 Dinoseb is a phenolic herbicide, highly toxic by ingestion and skin exposure, used in soybeans, vegetables fruits and nuts, citrus, grapes, and other field crops for the selective control of grass and broadleaf weeds (e.g., in corn) [13]. The solubility of dinoseb is reported as 52 mg/L [14]. Over a 10-year period, dinoseb was found to be one of three particularly persistent contaminants in Ontario wells supposed to find its way through spills of concentrated and dilute herbicide, drift during spraying, and from storm runoff [12]. Well water concentrations ranged from 0.05 to 5000 *μ*g/L and removal of dinoseb proved to be very difficult [14]. The reported half life of dinoseb ranges from 5 to 31 days in most circumstances [15].

Earlier photodegradation of C^14^ labeled dinoseb was done by exposing it to sunlight on growing bean foliage to determine the nature of the persisting residues [16]. Perchet et al. studied the degradation of dinoseb in presence of UV/TiO_2_ in nitramines and nitrophenol-contaminated waste water using ESI HPLC-MS [17]. Beside this study no detailed photocatalyzed degradation of dinoseb was reported. Therefore, we have studied a detailed degradation of dinoseb, in aqueous suspension of TiO_2_ under a variety of conditions such as types of TiO_2_, change in pH, catalyst loading, and substrate concentration and in the presence of different electron acceptors such as hydrogen peroxide (H_2_O_2_), potassium peroxodisulphate (K_2_S_2_O_8_), and potassium bromate (KBrO_3_) in presence of air. 


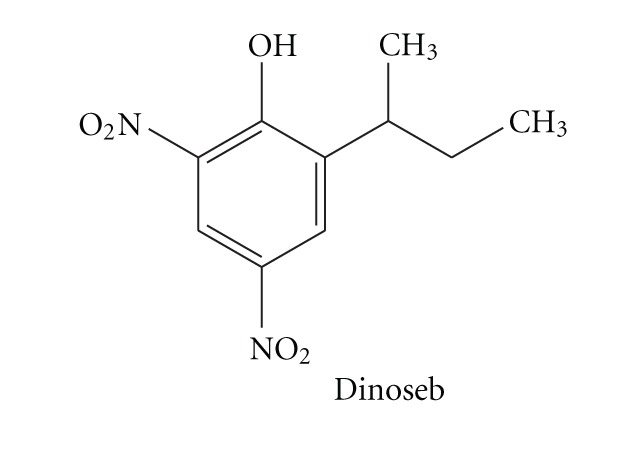


## 2. Experimental

### 2.1. Reagents and Chemicals

Analytical grade dinoseb was obtained from Reidel-de Haen (Sigma-Aldrich) and was used as such without any further purification. Solutions of dinoseb were prepared in double distilled water for irradiation experiments. Three commercially available TiO_2_ powders were used in this study, namely: (a) Degussa P25 (anatase: rutile 80: 20, specific BET 50 m^2^g^−1^, particle size 21 nm) [18]; (b) Hombikat UV100 (anatase, specific BET 250 m^2^g^−1^, particle size 5 nm) [19]; (c) PC500 (anatase, BET 320 m^2^g^−1^, particle size 5–10 nm) [20]. The other chemicals used in this study, such as sodium hydroxide, nitric acid, hydrogen peroxide, potassium persulphate, and potassium bromate are of reagent grade and were obtained from Merck.

### 2.2. Procedure

Stock solutions of the dinoseb with desired concentration were prepared in double distilled water. Experiments were carried out in an immersion well photo reactor made of Pyrex glass equipped with a magnetic bar, a water circulating jacket, and an opening for molecular oxygen. Required amount of the photocatalyst was then added and equilibration of the solution was assured by continuous stirring and purging of air for at least 15 minutes in the dark. Irradiations were carried out using 125 W medium pressure mercury lamp (Philips). The desired pH of the solution was maintained by adding dilute aqueous HNO_3_ (1 M) or NaOH (1 M) solutions before irradiation. Samples (5 mL) were collected before and at regular time interval during irradiation and analyzed after centrifugation. In order to determine the reproducibility of the results, at least triplicate runs were carried out for each condition for averaging the results, and the experimental error was found to be within ±5%.

### 2.3. Degradation of Dinoseb

The degradation of dinoseb was followed by measuring the change in absorption intensity at its *λ*_max_ (375 nm) using Shimadzu UV spectrophotometer (model 1601) and HPLC (Waters, 515 HPLC Pump and 2489 UV/Visible Detector, Column C-18, Eluent Acetonitrile: Water (70 : 30)).

### 2.4. Characterization of Intermediate Products

For the characterization of intermediate products, a solution of dinoseb (50 mg, 0.22 mM) in CH_3_CN/H_2_O mixture (1 : 1, 50 mL) was irradiated for different time intervals in the presence of TiO_2_ (Degussa P25, 1 g/L) using 312 nm light in a tubular photochemical reactor with constant stirring and bubbling of air in a quartz water-cooled reaction vessel. The solution was centrifuged to remove the catalyst, and clear solution was analyzed with GC-MS analysis. For GC-MS analysis Perkin Elmer Auto System XL (Gas Chromatograph) equipped with Perkin Elmer Turbo mass (Mass Spectrometry), operating temperature programmed (injection temperature 50°C for 1 min, which is raised to 250°C at the rate of 20°C min^−1^) in split mode of injection with helium as a carrier gas was used.

## 3. Results and Discussions

### 3.1. Photolysis of TiO_2_ Suspension Containing Dinoseb

**Figure 1:**
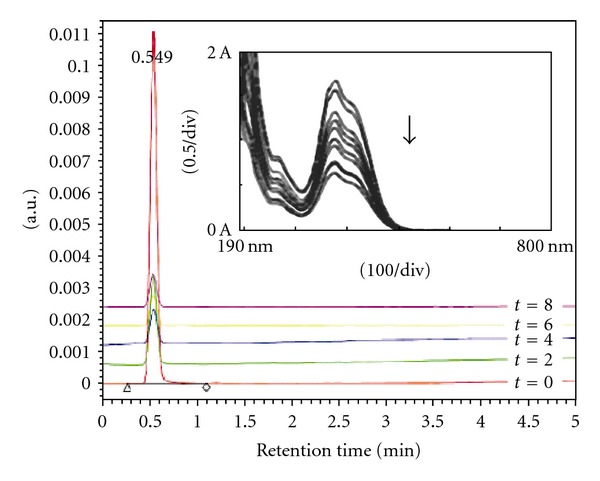
HPLC analysis of Dinoseb in an aqueous suspension of TiO_2_. Column C-18, eluent 70 : 30 (acetonitrile: water). Experimental conditions: 0.22 mM Dinoseb, *V* = 125 mL, photocatalyst TiO_2_ (Degussa P25, 1 gL^−1^), immersion well photo reactor, 125 W medium pressure Hg lamp, absorbance was followed at 375 nm, continuous purging of air and stirring, irradiation time: (a) 0 min; (b) 2 min; (c) 4 min; (d) 6 min; (e) 8 min. Inset shows decrease in absorbance on irradiation of aqueous solution of Dinoseb containing TiO_2_ under similar experimental conditions. Arrow shows the decrease in absorbance.

An aqueous solution of dinoseb (0.22 mM, 125 mL, pH 3.8) in the presence of TiO_2_ (Degussa P25, 1 gL^−1^) was irradiated with a 125 W medium pressure mercury lamp and the degradation was followed using both UV-Vis spectrophotometric and HPLC analysis techniques. Inset of the Figure 1 shows the decrease in absorption intensity as a function of irradiation time for the photocatalytic degradation of dinoseb, in an aqueous suspension of TiO_2_. In HPLC run, the starting material peak appearing at retention time *R*_*t*_ = 0.543 min was found to decrease on increasing irradiation time as shown in Figure 1. Blank experiments were carried out by irradiating the dinoseb solution using 125 W medium pressure mercury lamp in the absence of TiO_2_. The samples were collected at regular interval of time and analyzed by monitoring the change in concentration where no loss of dinoseb was observed. This may be due to the fact that in absence of photocatalyst the reactive species hydroxyl and superoxide radical are not formed which are likely to be the main attacking species for the photocatalytic oxidation.

For each experiment, the degradation rate constant for dinoseb was calculated from the linear regression of a plot of the natural logarithm of the compound concentration as a function of irradiation time, that is, pseudo first-order degradation kinetics using the formula given in (1):

(1) $$\frac{-d\left[C\right]}{dt}={kC}^{n},$$

*k* = rate constant, *C* = concentration of the pollutant, and *n* = order of reaction.

 The degradation rate for the decomposition of dinoseb for the pseudo first order reaction was calculated in terms of mmol L^−1 ^min^−1^.

### 3.2. Effect of Light Intensity

**Figure 2:**
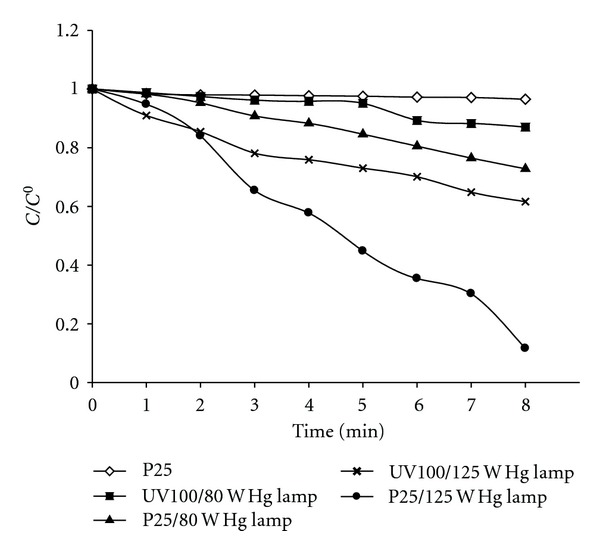
Effect of light intensity on degradation rate of Dinoseb. Experimental conditions: light intensity 80 and 125 W.

The incident light intensity is expected to be one of the rate-controlling parameters [21, 22]. In order to illustrate this effect, experiments were carried out using 80 and 125 W medium pressure Hg lamp. The light intensity, as measured by UV-light intensity detector (Lutron UV-340), was found to be 0.33 mW/cm^2^ (80 W) and 1.49 mW/cm^2^ (125 W). The corresponding decrease in concentration of dinoseb is represented in Figure 2. The degradation efficiency was found to be 24% with 80 W Hg lamp compared to 80% with 125 W Hg lamp.

These results may be explained based on the relatively less amounts of photons in the reaction system at low light intensity; on the other hand, at high intensity, photons present in excessive amounts lead to more reactive species generation and efficient destruction of pestanal; this proved that higher catalyst activity occurred under high light incident intensity. 

### 3.3. Comparison of Different Photocatalysts

**Figure 3:**
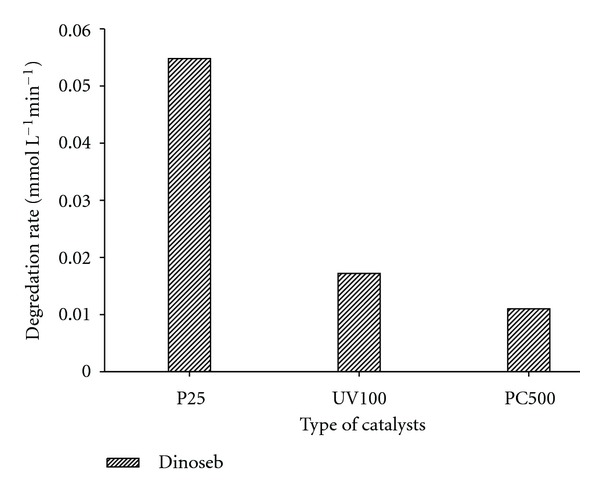
Comparison of degradation rate (change in absorption intensity versus irradiation time) of Dinoseb in the presence of different photocatalysts. Experimental conditions: photocatalysts: TiO_2_ Degussa P25 (1 gL^−1^), Sachtleben Hombikat UV100 (1 gL^−1^), and PC500 (1 gL^−1^).

The difference in efficiency of three types of photocatalysts, namely, Degussa P25, Hombikat UV100, and Millennium Inorganic PC 500 was tested for the degradation kinetics of dinoseb. The degradation of dinoseb was found to proceed much more rapidly in the presence of Degussa P25 as compared to other photocatalysts as shown in Figure 3 and the result is in good agreement with previously reported results [23]. 

The enhanced photocatalytic activity of Degussa P25 has been attributed to its mixed composition of rutile and anatase. The nanocrystallites of rutile having lower band gap energy being dispersed within the anatase matrix catch photons and enhance the electron-hole pair generation. Recombination is then prevented by electron transfer from rutile conduction band to electron traps in the anatase allowing the hole to move to the surface of the particle and react [4].

### 3.4. Effect of Catalyst Concentration

**Figure 4:**
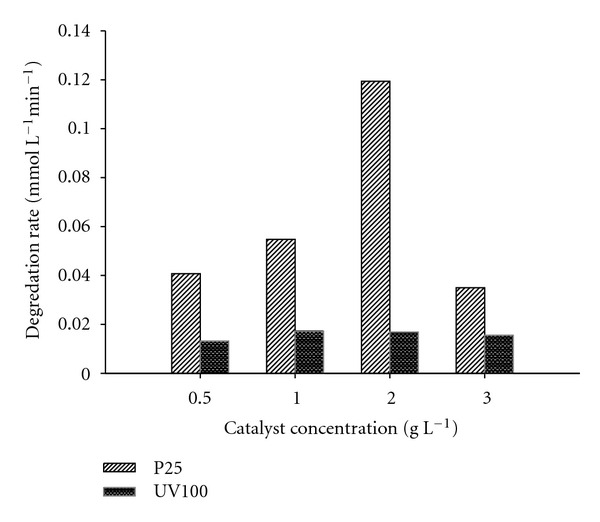
Influence of catalyst concentration on the degradation rate of Dinoseb. Experimental conditions: Photocatalysts TiO_2_ Degussa P25 and Hombikat UV100 (0.5, 1, 2 and 3 gL^−1^).

For any practical applications of photodegradation to waste water treatment, the optimum amount of catalyst is necessary in order to avoid excess catalyst and ensure total absorption of efficient photons. The effect of TiO_2_ amount on the degradation rate of dinoseb was investigated from 0.5 to 3 gL^−1^. The relationship between TiO_2_ loading (Degussa P25 and Hombikat UV100) and photodegradation rate of dinoseb is shown in Figure 4.

As a characteristic of heterogeneous photocatalysis, the degradation rate was found to increase with increase in catalyst concentration up to 2 gL^−1^ in case of P25 and 1 gL^−1^ in case of UV100. Further increase of catalyst concentration leads to slight decrease in the photodegradation rate of dinoseb and the result is in good agreement with number of studies reported earlier [2, 24, 25].

It is believed that both the number of photons absorbed as well as the solute molecules adsorbed increase with increase in number of TiO_2_ particles up to the optimum value. Any further increase in TiO_2_ concentration beyond optimum value may cause scattering and screening effects which reduces the specific activity of the catalyst [26]. The highly turbid suspension may prevent the catalyst farthest from being illuminated [27]. Higher amount of catalyst may lead to aggregation of TiO_2_ particles which may decrease the catalytic activity [28]. The optimum value of catalyst has been found to vary with different initial solute concentrations [29]. In all the following experiments, Degussa P25 was used as photocatalyst because of its better photocatalytic activity for the degradation of dinoseb.

### 3.5. Effect of pH

**Figure 5:**
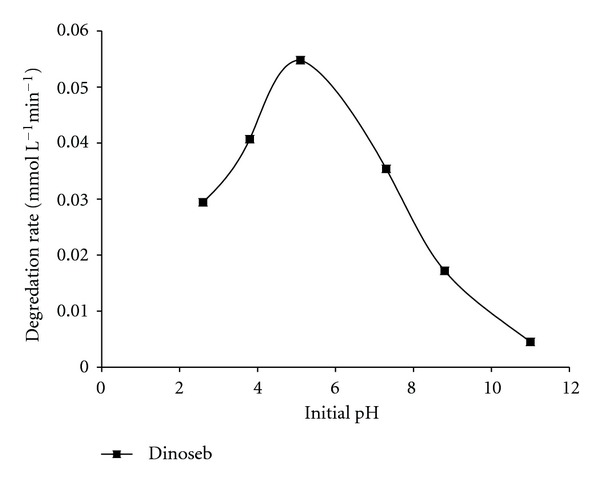
Influence of initial pH on the degradation rate of Dinoseb. Experimental conditions: reaction pH (2.6, 3.8, 5.1, 7.3, 8.8, and 11.0).

The difference in the pH values of different waste waters has a direct influence on the photocatalytic removal of the pollutants because pH determines the surface charge properties of the photocatalyst and therefore the adsorption behavior of the pollutant and also the size of the aggregates it forms. The zero point of charge (pH_zpc_) of P25 has been reported as 6.25 [30]. Hence above zero point charge (pH_zpc_), the particle surface is positively charged and vice versa. The reported pKa value of dinoseb is 4.62 [31]. As shown in Figure 5, the degradation of dinoseb was studied between pH 2 to 11, and efficient degradation was found at acidic pH of 5.1; the results are in good agreement with previously reported results on the degradation of 2,4-dinitrophenol in presence of ZnO [32]. The better degradation rate in acidic pH may be attributed on the basis of the fact that the structural orientation of the molecule is favoured for the attack of the reactive species under this condition.

### 3.6. Effect of Substrate Concentration

**Figure 6:**
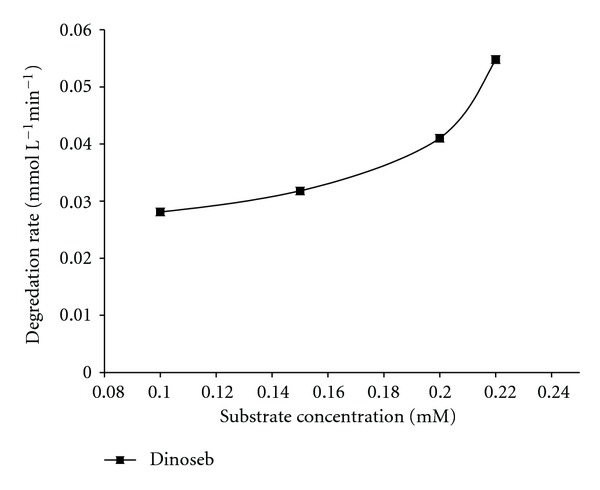
Influence of substrate concentration on the degradation rate of Dinoseb. Experimental conditions: substrate concentrations (0.10, 0.15, 0.20, and 0.22 mM).

From mechanistic as well as application point of view, the study of dependence of degradation rate on substrate concentration is important. The degradation of dinoseb was studied between 0.1 mM to 0.22 mM.  Figure 6 shows that the degradation rate increases with increase in substrate. This may be justified by the fact that at lower substrate concentration, with fixed amount of TiO_2_, all the catalytic sites are not occupied leading to lower degradation rate. With increase in the substrate concentration up to optimum value, more and more catalytic sites get occupied which leads to progressive increase in degradation rate. The result is in good agreement with previously reported studies [4, 33–35].

### 3.7. Effect of Electron Acceptors

**Figure 7:**
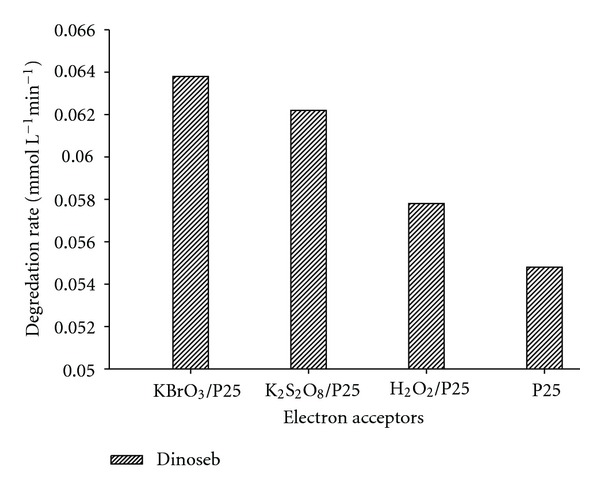
Comparison of degradation rate of Dinoseb in the presence of various electron acceptors like H_2_O_2_, KBrO_3_, and K_2_S_2_O_8_. Experimental conditions: electron acceptor: H_2_O_2_ (10 mM), KBrO_3_ (3 mM), and K_2_S_2_O_8_ (3 mM).

The major energy wasting step that limits the achievable quantum yield in photocatalysis is the undesired electron/hole recombination. One way to overcome this is to add other (irreversible) electron acceptors to the reaction mixture. The effect of electron acceptors such as potassium bromate, potassium peroxodisulphate, and hydrogen peroxide in presence of air on the degradation kinetics of dinoseb has been investigated and the results are depicted in Figure 7. All employed additives showed effective electron accepting power than molecular oxygen as is expected from their respective one-electron reduction potential E(O_2_/O_2_^•−^) = −155 mV, E(H_2_O_2_/HO^•^) = 800 mV, E(BrO_3_^−^/BrO_2_^•^) = 1150 mV, and E(S_2_O_8_^2−^/SO_4_^•−^) = 1100 mV [36]. The reason for enhanced degradation of dinoseb by the addition of these additives may be explained by the formation of strong oxidizing radicals according to the following reactions:

(2) $${\text{H}}_{2}{\text{O}}_{2}+{\text{e}}_{\text{CB}}^{-}\to {\text{OH}}^{•}+{\text{OH}}^{-},$$

(3) $${{\text{S}}_{2}{\text{O}}_{8}}^{2-}+{\text{e}}_{\text{CB}}^{-}\to {{\text{SO}}_{4}}^{2-}+{{\text{SO}}_{4}}^{•-},$$

(4) $${{\text{SO}}_{4}}^{•-}+{\text{H}}_{2}\text{O}\to {{\text{SO}}_{4}}^{2-}+{\text{OH}}^{•}+{\text{H}}^{+},$$

(5) $${\text{Br}{\text{O}}_{3}}^{-}+{2\text{H}}^{+}+{\text{e}}_{\text{CB}}^{-}\to {\text{Br}{\text{O}}_{2}}^{•}+{\text{H}}_{2}\text{O},$$

(6) $${\text{Br}{\text{O}}_{3}}^{-}+{6\text{H}}^{+}+{6\text{e}}_{\text{CB}}^{-}\to \left[\text{Br}{\text{O}}_{2},\text{HOBr}\right]\to {\text{Br}}^{-}+{3\text{H}}_{2}\text{O}.$$

Addition of potassium bromate showed pronounced effect for the degradation of dinoseb compared to the other additives. This has been attributed to the greater number of electrons it reacts as shown in (6).

### 3.8. Photocatalysis of Dinoseb for Product Analysis

**Figure 8:**
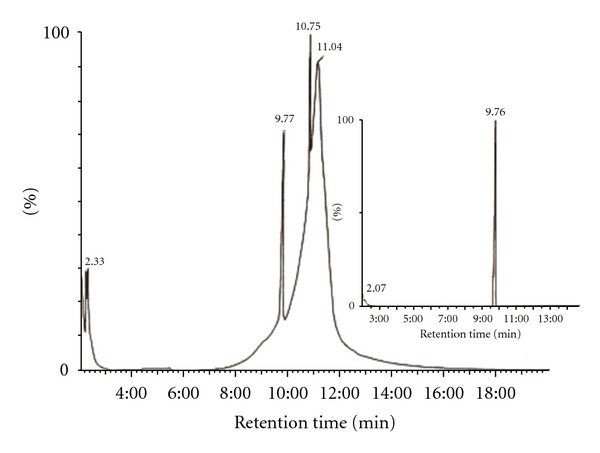
GCMS analysis of an irradiated Dinoseb in 1 : 1 acetonitrile/water mixture in the presence of TiO_2_ for 8 h. Inset shows GCMS analysis of Dinoseb before irradiation.

**Figure 9:**
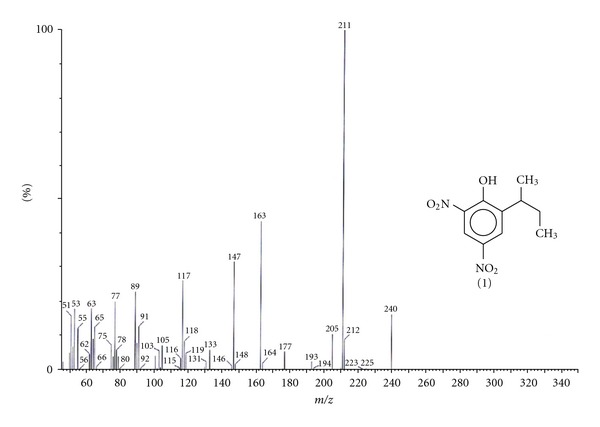
Mass fragmentation pattern of unilluminated Dinoseb appearing at *R*_*t*_ 9.76.

An attempt was made to identify the intermediates products formed during the photooxidation through GC-MS analysis technique. The analysis of an irradiated mixture of dinoseb in the presence of TiO_2_ in 1 : 1 acetonitrile/water mixture for 8 h showed formation of two peaks appearing at *R*_*t*_ 11.04 and 10.75 min in addition to the unchanged starting material at *R*_*t*_ 9.77 min as shown in Figure 8. The analysis of dinoseb prior to irradiation showed a single peak appearing at *R*_*t*_ 9.76 min as shown in inset of Figure 8. The mass fragmentation shown in Figure 9 is comparable with that reported in the GC-MS library for dinoseb.

**Figure 10:**
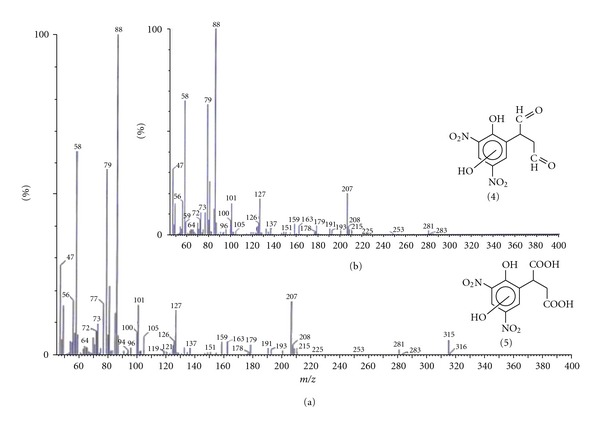
Mass fragmentation pattern of the two product peaks appearing at (a) *R*_*t*_ 11.04 and (b) *R*_*t*_ 10.75.

**Scheme 1:**
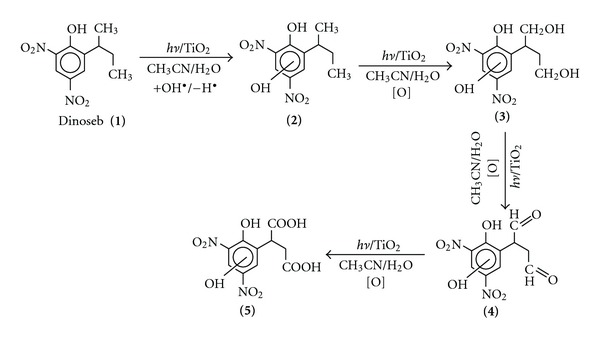
Probable pathway for the degradation of Dinoseb catalyzed by TiO_2_ in the presence of UV light.

 The products have been characterized on the basis of molecular ion and mass fragmentation pattern shown in Figure 10. The formation of the two products could be understood in terms of the pathway shown in Scheme 1. Dinoseb may undergo hydroxyl radical insertion followed by loss of hydrogen atom to give the product **2** which may intern undergo sequential oxidative reaction of terminal methyl groups to give first aldehyde **4 **(*R*_*t*_ 11.04) and then dicarboxylic acid derivative **5 **(*R*_*t*_ 10.75). It is pertinent to mention here that hydroxyl radical insertion reaction in aromatic ring and oxidative conversion of methyl into carboxylic group has been reported in the literature under similar reaction conditions [37, 38].

## 4. Conclusion

The results of this study clearly indicate that TiO_2_ can efficiently catalyse the photo degradation of the pollutants in the presence of light and oxygen. All the parameters have been found to show pronounced effect on the degradation kinetics of dinoseb. Characterisation of intermediate products formed during the photooxidation process using GCMS analysis indicates the formation of hydroxyl insertion product and side chain oxidation of alkyl group. Formation of these products in such reactions gives mechanistic information of surface reactions of the organic compounds.
